# The Vocal Repertoire of the African Penguin (*Spheniscus demersus*): Structure and Function of Calls

**DOI:** 10.1371/journal.pone.0103460

**Published:** 2014-07-30

**Authors:** Livio Favaro, Laura Ozella, Daniela Pessani

**Affiliations:** Department of Life Sciences and Systems Biology, University of Turin, Turin, Italy; University of Pavia, Italy

## Abstract

The African Penguin (*Spheniscus demersus*) is a highly social and vocal seabird. However, currently available descriptions of the vocal repertoire of African Penguin are mostly limited to basic descriptions of calls. Here we provide, for the first time, a detailed description of the vocal behaviour of this species by collecting audio and video recordings from a large captive colony. We combine visual examinations of spectrograms with spectral and temporal acoustic analyses to determine vocal categories. Moreover, we used a principal component analysis, followed by signal classification with a discriminant function analysis, for statistical validation of the vocalisation types. In addition, we identified the behavioural contexts in which calls were uttered. The results show that four basic vocalisations can be found in the vocal repertoire of adult African Penguin, namely a *contact call* emitted by isolated birds, an *agonistic call* used in aggressive interactions, an *ecstatic display song* uttered by single birds, and a *mutual display song* vocalised by pairs, at their nests. Moreover, we identified two distinct vocalisations interpreted as begging calls by nesting chicks (*begging peep*) and unweaned juveniles (*begging moan*). Finally, we discussed the importance of specific acoustic parameters in classifying calls and the possible use of the source-filter theory of vocal production to study penguin vocalisations.

## Introduction

Establishing a comprehensive classification of bird vocalisations is important for avifaunal surveys, allows comparisons between species and individuals [1], and also contributes to planning effective management and conservation strategies [2]. Indeed, vocalisations have the potential to provide a variety of information about bird sex, age, behavioural state, condition, and relationships with surrounding animals [3]. Moreover, avian vocalisations are important to establish phylogenetic relationships and in the discovery of new species [1].

Bird calls are produced through the syrinx [4], which manifests several anatomical differences compared to the mammalian larynx. In particular, the syrinx is located at the base of the trachea, while the mammalian larynx sits above it [5]. Moreover, the syrinx is a two-part organ where the sound is produced by an independent set of muscles, along with membranes at the right and left sides [6]. Unlike mammalian vocal folds, this anatomical configuration allows many birds, including penguins, to produce two independent signals simultaneously [7]. However, syringeal constriction functionally resembles the larynx in mammalian phonation, and the trachea can act as a filter to dump or accentuate certain frequencies, creating formant peaks [5], thus modifying the spectrographic structure of calls. For these reasons, the source-filter theory of mammalian vocal production [8], [9] has also been used to explain the acoustic output of many avian vocalisations [10], [11]. Moreover, regarding birds, it has been demonstrated that the energy distribution in the spectrum can be affected by modifications of the pharyngeal cavity and the oesophagus [12].

Penguins have three basic call types: *contact calls*, *agonistic calls*, and *display songs* [13]. *Display songs* can be further divided into *ecstatic display songs* (uttered by single birds) and *mutual display songs* (uttered by pairs). Moreover, penguin songs have smallest units, namely syllables, which may be combined into phrases [13]. Historically, penguins' vocal behaviour has been extensively investigated in Antarctic, sub-Antarctic, and Australian species, which use *display songs* for recognition between mates and between chicks and parents [14]. In particular, Aubin *et al.* [7] demonstrated that non-nesting species, such as the Emperor Penguin (*Aptenodytes forsteri*) and the King Penguin (*Aptenodytes patagonicus*), use the two-voices system as principal mean to identify each other. Further, Jouventin and Aubin [15] showed that in nesting species, such as the Adélie Penguin (*Pygoscelis adeliae*) and the Gentoo Penguin (*Pygoscelis papua*), the pitch of the song and the frequency and relative values of harmonics are the main cues for individual recognition. Conversely, much less research effort has been directed toward the study of the vocal behaviour of the temperate and equatorial species of the genus *Spheniscus*.

The African Penguin is highly social and breeds on islands and coastal areas of South Africa and Namibia [16]. This species makes use of several distinctive vocalisations for intra-specific communication [17]. However, currently available descriptions of the vocal repertoire of *S. demersus* (summarised in Table S1) are mostly limited to basic descriptions of calls. Thumser and Ficken [18] reported five distinct vocalisations made by two captive populations of African Penguin. These authors also measured some temporal parameters and three frequency parameters on two vocalisation types, that they labelled as *haw* and *bray*, and which correspond to the *ecstatic display song* and *mutual display song*, respectively, described by Eggleton and Siegfried [17] and Jouventin [13]. They also published spectrographic representations of these two calls. Overall, the data presented by Thumser and Ficken [18] are very limited as recordings were obtained from a restricted number of birds and acoustic signals, and only took place during the breeding season (Table S1). Moreover, the lack of acoustic measurements on the majority of the call types does not provide an adequate structural and quantitative description of the entire vocal repertoire of this species.

The African Penguin is seriously threatened, because the total population has dramatically decreased in recent years to less than 75–80,000 mature individuals [19]. The decline is mainly due to loss of habitat, reduction of fish stocks, environmental pollution (including oil spills), and egg collection [16], [20], [21]. For these reasons, this species is currently included in CITES (Convention on International Trade in Endangered Species of Wild Fauna and Flora) Appendix II, in CMS (Convention on the Conservation of Migratory Species of Wild Animals) Appendix II, and its classification within the Red List of Threatened Species of the IUCN (International Union for Conservation of Nature) was changed from “Vulnerable” to “Endangered” in 2010.

Animal sound recording and analysis technology have greatly advanced in recent years [22]. Technological improvements now enable the implementation of extended audio recordings, the automation of the process of signal analysis, and the measurement of a variety of spectral and temporal acoustic parameters with a limited computational effort [22], [23]. Recent studies of animal vocalisations are also focussed on statistically quantifying the similarities or differences between acoustic signals by means of multivariate statistical techniques [24] or mathematical computational approaches [25], in order to eliminate subjectivity.

Here, we examined the vocalisations of the African Penguin by collecting audio and video recordings from a captive colony in Italy. Firstly, we categorised vocal signals by visual inspection of spectrograms, and by matching the vocalisations to the behavioural contexts in which they were produced. Subsequently, we measured a variety of spectral and temporal acoustic parameters that we used for statistical validation of the vocal categories. We aimed to provide a detailed description of the entire vocal repertoire of this species and to standardise terminology for use in future studies. Finally, we discuss the importance of the different acoustic parameters in characterizing the vocal types.

## Methods

### Ethics Statement

The study complies with all applicable Italian laws, with the Guidelines for the Treatment of Animals in Behavioural Research and Teaching [26] and with the Ethical Guidelines for the Conduct of Research on Animals by Zoos and Aquariums [27]. The research was carried out with permission from ZOOM Torino (www.zoomtorino.it), Cumiana, Italy (44°56′N, 7°25′E). This zoological institution has rigorous standards for animal welfare and is accredited by the EAZA (European Association of Zoos and Aquaria) and UIZA (Unione Italiana Giardini Zoologici e Acquari). Since all recording procedures were non-invasive and did not cause any disturbance to the animals during their normal daily activity, this study does not fall in any of the categories for which approval of an ethic committee is required by Italian laws.

### Penguins and recordings

Vocalisations and associated behaviours were collected from a captive colony of 48 African Penguins at ZOOM Torino, Italy. The composition of the colony in December 2011 was 15 males, 17 females, 8 juveniles (3 to 12 months), and 8 nesting chicks (<3 months). Penguins were housed in an outdoor communal exhibit of 1500 m^2^, including a pond of 120 m^2^ (maximum depth 3 m) and each penguin was identified with wing tag. Data were collected using the all-occurrence sampling method [28] over 24 non-consecutive days from September to October 2010, and 80 non-consecutive days from August to December 2011. All recordings were collected from outside the exhibit, without any manipulation of the penguins and without the use of playback stimuli.

Acoustic recordings were carried out with a RØDE NTG-2 semi-directional microphone (frequency response 20 Hz to 20 kHz, max SPL 131dB) connected to a TASCAM DR-680 digital recorder (48 kHz sampling rate). During recording sessions, the microphone was mounted on a RØDE PG2 Pistol Grip to reduce handling noise and was placed at a distance of 1–10 m from the vocalising penguins. Segments containing acoustic recordings were saved in WAV format (16-bit amplitude resolution) and stored on a secure digital (SD) memory card for later analyses. Simultaneously to acoustic recordings, we monitored the penguins' activities using a JVC Everio GZ-MG330 camcorder with 35× Optical Zoom for a detailed identification of the behavioural contexts in which calls were produced. In particular, we identified behaviours according to the ethogram for this species provided by Eggleton and Siegfried [17].

### Spectrographic analysis

We analysed 271 hours of audio recordings. For each audio file, the waveform and the FFT (Fast Fourier Transform) spectrogram were generated with the Praat v. 5.3.39 [29] sound editor window, using a customised spectrogram setting [view range = 0 to 10000 Hz, window length = 0.02 s (Gaussian window shape, −3 dB bandwidth 65 Hz), number of time steps = 1000, number of frequency steps = 500 (frequency resolution 20 Hz), dynamic range = 50 dB]. The visual examinations of spectrograms allowed us to identify 1171 vocalisations that we subsequently divided into macro vocal categories. In particular, we identified: a *contact call* (n = 331), an *agonistic call* (n = 138), an *ecstatic display song* (n = 179), a *mutual display song* (n = 293), and a nesting chicks' vocalisation, namely the *begging peep* (n = 160). Moreover, we were able to distinguish an additional vocal type, namely the *begging moan*, emitted as a food request by juveniles (n = 70). Since the *begging peep* and *begging moan* were uttered by penguins in long sequences, in order to avoid the risk of pseudo-replication, we only considered one signal from each sequence. From this original dataset, we further selected 391 good quality calls [*contact call* = 36 (contributed by 13 individuals; 2.8±2.9 calls per individual, mean ± standard deviation), *agonistic call* = 47 (contributed by 11 individuals; 4.6±4.1 calls per individual), *ecstatic display song* = 83 (contributed by 9 individuals; 5.8±4.6 calls per individual), *mutual display song* = 39 (contributed by 13 individuals; 3.0±2.5 calls per individual), *begging moan* = 57 (contributed by 3 individuals; 19.0±12.3 calls per individual), *begging peep* = 129 (contributed by 4 individuals; 32.2±10.1 calls per individual)] on which to collect acoustic measurements. The large number of vocalisations excluded in this second phase (66.61%) was mainly due to the difficulties encountered during field recordings. In particular, 114 *mutual display songs* were discarded because of overlapping songs between mates (usually vocalising within the nest) and 36 *ecstatic display songs* were discarded because of overlapping between males vocalising at the same time in different areas of the exhibit. Regarding the rest of the excluded signals, they were not considered as being acceptable for the measurement of acoustic parameters because they showed an insufficient signal-to-noise ratio of the pitch. Indeed, although our recordings were collected in an outdoor enclosure without severe reverberation and sound distortion effects that characterise many indoor exhibits, a high level of background noise was present, mainly due to the high number of visitors.

### Acoustic analysis

For each selected vocalisation, we measured 15 spectral and temporal acoustic parameters (Table 1) using semi-automated procedures with a custom-built program [30], [31] in Praat v. 5.3.39 [29]. We used descriptors related to the ‘source’ component of calls (F0). Moreover, we considered the energy quartiles as filter-related vocal parameters but we did not measure formant peaks, as whilst they were evident in certain call types, they were only weakly detectable in others, to the extent of being unrecognisable, for example, in chicks' vocalisations. This decision was made in order to only include variables that could be collected from all signals.

**Table 1:** List and abbreviations of the acoustic parameters measured on each call.

Abbreviation	Parameter
F0 start (Hz)	Frequency value of F0 at the start of the call
F0 end (Hz)	Frequency value of F0 at the end of the call
F0 mean (Hz)	Mean F0 frequency value across the call
F0 min (Hz)	Minimum F0 frequency value across the call
F0 max (Hz)	Maximum F0 frequency value across the call
F0 range	F0 max - F0 min
F0AbsSlope (Hz/s)	F0 mean absolute slope
FM rate (s-1)	Number of complete cycles of F0 modulation per second
Jitter (%)	Mean absolute difference between frequencies of consecutive F0 periods divided by the mean frequency of F0
Shimmer (%)	Mean absolute difference between the amplitudes of consecutive F0 periods divided by the mean amplitude of F0
Q25% (Hz)	Frequency value at the upper limit of the first quartiles of energy
Q50% (Hz)	Frequency value at the upper limit of the second quartiles of energy
Q75% (Hz)	Frequency value at the upper limit of the third quartiles of energy
AM rate (s-1)	Number of complete cycles of amplitude modulation per second
Dur (s)	Duration of the call

We extracted the F0 contour of each call using a cross-correlation method [Sound: To Pitch (cc) command]. Depending on the acoustic characteristics of each vocal type, we used a time step of 0.01–0.02 s, a pitch floor of 150–1000 Hz, and a pitch ceiling of 350–2500 Hz. From each extracted F0 contour, we obtained the frequency value of F0 at the start (F0Start) and at the end (F0End) of the call; the F0 range (F0Range); the mean (F0Mean), minimum (F0Min) and maximum (F0Max) F0 frequency values across the call. In addition, we obtained the F0 mean absolute slope (F0AbsSlope), which is a measure for the average local variability in F0, by computing the average slope between adjacent points on the pitch curve. Furthermore, we measured the number of complete cycles of fundamental frequency modulation per second (FM rate), and we quantified the number of complete cycles of amplitude modulation per second (AM rate). We also calculated Jitter [the mean absolute difference between frequencies of consecutive F0 periods divided by the mean frequency of F0 (Jitter (local) command)] and Shimmer [the mean absolute difference between the amplitudes of consecutive F0 periods divided by the mean amplitude of F0 (Shimmer (local) command)] values. Jitter and Shimmer are measures of the cycle-to-cycle variations of fundamental frequency and amplitude, respectively [32]–[34]. For a detailed description of the algorithms used by Praat to calculate Jitter and Shimmer, please refer to Boersma [35]. These parameters have been widely used for the study of pathological disorders of the human voice [36], speaker recognition [37] and, above all, in the analysis of arousal and valence in human and non-human mammal vocalisations [38]–[40]. Finally, we measured the frequency values at the upper limit of the first (Q25%), second (Q50%) and third (Q75%) quartiles of energy, using a linear amplitude spectrum, and we included the total duration of each call (Dur) in the analyses.

Finally, on the *ecstatic display song*, in order to describe the structural proprieties of this complex call, we identified syllables (according to the terminology used by Jouventin [13]) and we measured the mean number of syllables per song, and the sum of all inter-syllable intervals (s). However, we limited the spectral analysis to the longest syllable of the song.

### Statistical analysis

All analyses were performed in SPSS v. 20 (SPSS, Inc. 2010). Firstly, we log-transformed our data as they significantly deviated from a normal distribution (Kolmogorov-Smirnov test). In addition, to meet the assumption of independence between the acoustic variables, we performed a Principal Component Analysis (PCA) using an orthogonal varimax rotation [41]. The PCA reduce the original set of acoustic measurements to a new set of uncorrelated principal components (PCs). PCs showing eigenvalues >1 were used to classify vocalisations with a stepwise, cross-validated (leave-one-out) discriminant function analysis (DFA). In particular, we entered the type of call as the grouping variable and the PCs scores as predictors. Finally, we used the Wilks' Lambda (λ) method to measure how well each function separated cases into groups.

## Results

### Spectrographic classification of the vocal repertoire

**Figure 1:**
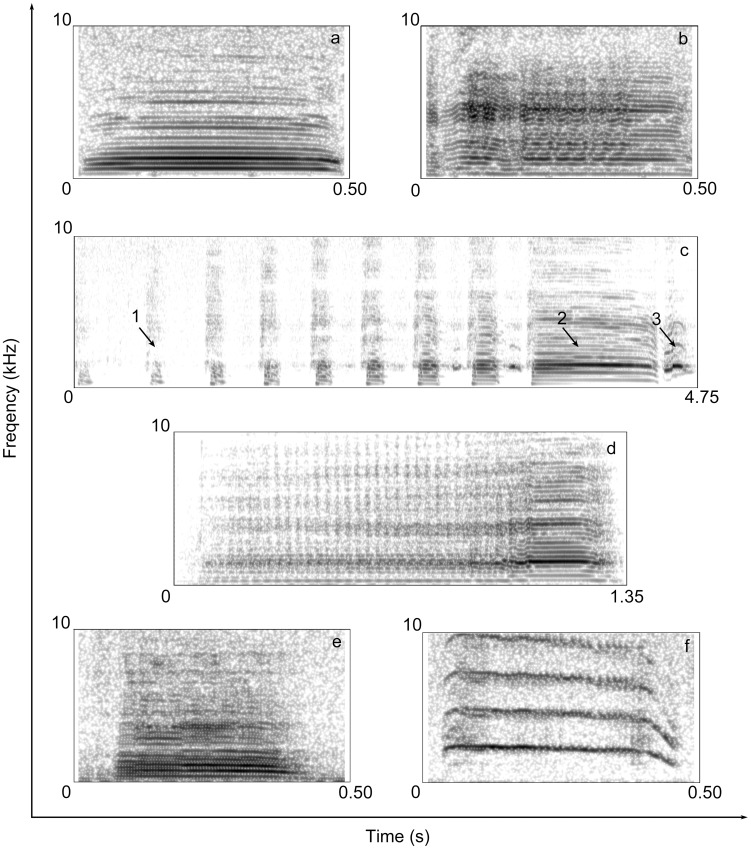
Spectrographic representation of the vocal categories identified in the repertoire of the African Penguin. *Contact call* (a), *agonistic call* (b), *ecstatic display song* (c: arrows indicate short initial syllables 1, longest syllable 2, inspiration syllable 3), *mutual display song* (d), *begging moan* (e), *begging peep* (f). Spectrograms were generated in Praat using a Gaussian window shape, window length = 0.02 s, number of time steps = 1000, number of frequency steps = 500, dynamic range = 50 dB.

A spectrographic representation of the vocal categories identified by visual inspection of spectrograms is presented in Figure 1. Below, we describe the call types in detail, including the contexts of emission.

*Contact call* (Figure 1a; Video S1)
  The *contact call* is a short call (0.58±0.18 s) consisting of a single utterance. The vocalisation has a clear harmonic structure and it is possible to observe from the spectrogram that the output signal is filtered by the resonant properties of the vocal tract. During emission of this call, the beak is half-open and the emitter stands up, extending the neck upwards as much as possible. We recorded this vocalisation in juveniles and adults of both sexes.
*Agonistic call* (Figure 1b; Video S2)
  Similarly to the *contact call*, the *agonistic call* is a single utterance that shows a clear harmonic structure and a short duration (0.44±0.15 s). This vocalisation has high Jitter (2.12±1.09%) and Shimmer (14.47±3.87%) values compared to the other types of call. During utterance of this call, the birds stand up and extend the neck towards the recipient of the aggression. The *agonistic call* was recorded in both juveniles and adults.
*Ecstatic display song* (Figure 1c; Video S3)
  The *ecstatic display song* is the longest (5.04±4.17 s) and loudest vocalisation in the vocal repertoire of this species. Penguins emitted this utterance resting with their feet apart, their neck and beak facing upward, and their wings arranged horizontally. The song is composed of a sequence of vocal units or syllables (mean number per call = 12.3±1.3; sum of inter-syllable intervals across the call = 3.57±1.23 s) combined in a phrase. This vocalisation begins with a sequence of short syllables (mean duration of each syllable = 0.18±0.05 s; Figure 1c – indicated by the arrow 1) during which the keeled sternum moves upwards and downwards and culminates with the emission of a long syllable (mean duration = 1.14±0.33 s; Figure 1c – indicated by the arrow 2) during which the sternum remains upwards. Occasionally, we observed changes in the general pattern of this vocalisation with the presence of two long syllables per call, as well as calls without the emission of the longest syllable. Finally, we identified a third type of syllable (mean duration = 0.38±0.12 s; Figure 1c – indicated by the arrow 3) produced during the inhalation phase that follows the emission of the longest unit.
*Mutual display song* (Figure 1d; Video S4)
  This utterance begins with pulsed noises and ends with a clear low-pitched harmonic structure (F0 mean = 285±21 Hz). During phonation, the body is usually horizontal, the neck is extended as much as possible, and the beak is wide open. The mean duration of the *mutual display song* recorded in this study was 1.45±0.29 s and we measured high Jitter (4.30±1.32%) and Shimmer (17.44±2.68%) values, comparable to those observed in the *agonistic call*.
*Begging moan* (Figure 1e; Video S5)
  The *begging moan* was only emitted by juveniles (3 to 12 months of age). This vocal signal shows a clear harmonic structure and a short duration (0.27±0.11 s). Juvenile penguins emitted long sequences of 1 to 10 *begging moans*, but they immediately stopped calling when they were fed, or when the parent moved away. During utterance, juveniles performed quick lateral movement with their heads.
*Begging peep* (Figure 1f; Video S6)
  The *peep* is a begging call emitted by chicks (<3 months of age) inside the nest either in the presence or absence of their parents. The average duration of a single *peep* recorded in this study was only 0.36±0.07 s but this call was repeated by chicks in long sequences lasting for several minutes, until they were fed. The peep is a high-pitched vocalisation (F0 mean = 1851±199 Hz), and we observed harmonic frequencies of up to 17 kHz.

### Statistical classification of the vocal repertoire

Descriptive statistics of vocal parameters for each vocalisation type are presented in Table 2. The original set of 15 acoustic parameters was transformed by the PCA into three PCs showing eigenvalues >1 (Table 3) that accounted for 91.33% of the total variance (PC1 = 60.0%, PC2 = 14.59%, PC3 = 9.64%, PC4 = 7.03%). In particular, PC1 was highly correlated (r>0.70) with F0 values (source-related parameters), PC2 with Jitter and Shimmer (parameters related to F0 variation) and call duration, PC3 with the upper limit of the first, second and third quartiles of energy (filter-related parameters), and PC4 with both FM rate and AM rate.

**Table 2:** Descriptive statistics for each vocal category.

	Vocal category					
Acoustic parameter	*Contact call* (n = 36)	*Agonistic call* (n = 47)	*Ecstatic display song* (n = 83)	*Mutual display song* (n = 39)	*Begging moan* (n = 57)	*Begging peep* (n = 129)
F0 start (Hz)	258±34	251±36	272±25	264±26	229±27	1731±236
F0 end (Hz)	228±36	227±35	228±25	258±24	198±38	1425±211
F0 range (Hz)	70±26	73±26	55±23	58±22	45±16	619±247
F0 mean (Hz)	282±25	272±30	267±18	285±21	275±35	1851±199
F0 min (Hz)	226±35	221±30	227±24	248±22	195±31	1395±102
F0 max (Hz)	295±25	294±32	282±22	306±24	240±37	2015±245
F0AbsSlope (Hz/s)	217±79	384±117	96±77	132±44	271±171	2767±1096
Jitter (%)	0.90±0.33	2.12±1.09	0.51±0.62	4.30±1.32	1.41±0.96	5.11±2.01
FM rate (s-1)	1.84±1.14	3.54±1.83	2.68±1.73	6.16±1.08	1.80±1.78	3.63±1.87
Shimmer (%)	8.90±4.07	14.47±3.87	6.24±2.60	17.44±2.68	14.40±4.11	16.61±3.87
Q25 (Hz)	544±181	448±164	861±293	420±182	334±204	321±557
Q50 (Hz)	811±186	820±242	1244±205	848±313	630±271	778±671
Q75 (Hz)	1310±692	1500±406	1645±202	1443±311	1082±315	2453±1051
AM rate (s-1)	14.15±4.38	18.75±4.72	16.64±4.19	26.51±2.45	14.67±6.58	26.53±4.68
Dur (s)	0.58±0.18	0.44±0.15	5.04±4.17	1.45±0.29	0.27±0.11	0.36±0.07

Table shows mean values ± standard deviation.

**Table 3:** Results of the principal component analysis with varimax rotation.

	Principal Component			
Acoustic parameter	1	2	3	4
F0 start	0.959*	0.167	−0.092	0.160
F0 end	0.941*	0.189	−0.083	0.204
F0 range	0.928*	0.213	−0.046	0.079
F0 mean	0.961*	0.181	−0.074	0.170
F0 min	0.946*	0.183	−0.084	0.197
F0 max	0.961*	0.191	−0.077	0.168
F0 AbsSlope	0.814*	0.502	−0.148	0.054
FM rate	0.040	0.157	−0.008	0.871*
Jitter	0.453	0.660	−0.170	0.458
Shimmer	0.205	0.817*	0.013	0.437
Q25	−0.549	−0.214	0.732*	−0.123
Q50	−0.198	−0.192	0.925*	−0.103
Q75	0.597	0.017	0.731*	0.059
AM rate	0.461	0.048	0.145	0.709*
Dur	−0.225	−0.873*	0.271	0.105

The table shows factor loadings of the acoustic parameters on the principal components showing eigenvalues >1 (PC1–PC4) extracted from the PCA.

Note:

*heaviest factor loadings (*r*>0.70).

**Figure 2:**
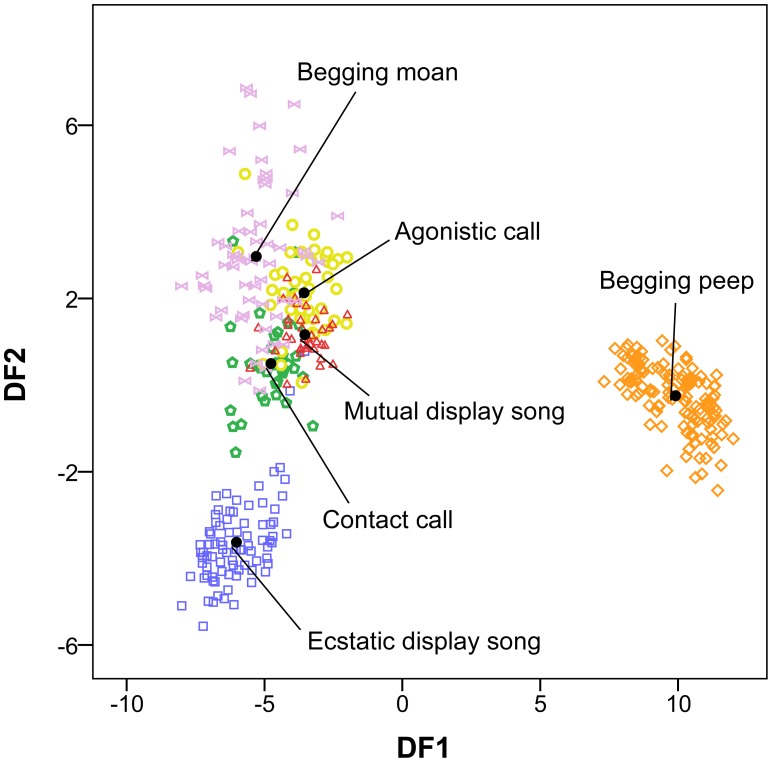
Plot of the discriminant scores generated by the first two discriminant functions to classify vocalisations of the African Penguin. Black dots are the centroids of the vocal categories.

The stepwise, cross-validated DFA correctly classified 90.5% of the vocal signals according to the predicted vocal categories that we assigned by inspection of spectrograms. The analysis generated four discriminant functions which revealed a highly significant difference between call types (Wilks' λ DF1/4 = 0.002, χ^2^ = 2446.73, p<0.001; Wilks' λ DF2/4 = 0.088, χ^2^ = 934.53, p<0.001; Wilks' DFλ 3/4 = 0.519, χ^2^ = 252.48, p<0.001; Wilks' λ DF4 = 0.985, χ^2^ = 5.93, p<0.05). The six vocal categories form distinctive clusters in the space defined by discriminant functions 1 and 2 (Figure 2). The percentage of correct assignment of each signal to the predicted vocal category is presented in Table 4.

**Table 4:** Classification results of the stepwise cross-validated (leave-one-out) discriminant function analysis.

Vocal category	Predicted vocal category						
	*Contact call*	*Agonistic call*	*Ecstatic display song*	*Mutual display song*	*Begging moan*	*Begging peep*	Total (%)
*Contact call*	77.8*	13.9	0	0	8.3	0	100
*Agonistic call*	8.5	74.5*	0	6.4	10.6	0	100
*Ecstatic display song*	0	0	97.6*	2.4	0	0	100
*Mutual display song*	0	0	0	100*	0	0	100
*Begging moan*	12.3	14	0	0	73.7*	0	100
*Begging peep*	0		0	0	0	100*	100

Note:

*percentage of correct classification for the predicted vocal category.

## Discussion

Here we provide the first detailed acoustic analysis of the entire vocal repertoire of the African Penguin by selecting and analysing 391 vocal signals collected from a captive colony. Firstly, we categorised the vocalisations based on the visual inspection of spectrograms and behavioural contexts of vocal emissions. According to the general categorisation of penguin calls provided by Jouventin [13], we were able to identify four different call types uttered by adult African Penguins and two begging vocalisations [42] emitted by nesting chicks and unweaned juveniles, respectively. In particular, we found a *contact call* produced by single members of the colony when visually isolated from the rest of the group or from the partner. Specific behaviours associated with this vocalisation are the “look around” and “slander walk” [17]. According to Jouventin [13], we suggest that this vocalisation enables isolated penguins to locate other members of the colony. Moreover, we report an *agonistic call* uttered during fights or when intruding penguins approached a nest already occupied by a pair. It was also produced by penguins that were chasing away other members of the colony. This vocalisation was frequently preceded or followed by a peck from the emitter. We occasionally recorded *agonistic calls* during the feeding sessions, especially when penguins were gathered together and there was a high level of arousal in the group. In this case, we suggest that this call was being posed as an acoustic threat. Associated with the *agonistic call* are the specific behaviours of “point”, “gape” and “peck” [17]. This utterance is perceived by human listeners as being rough and hoarse, probably due to the high Jitter and Shimmer values. The *ecstatic display song* is a call produced during the ecstatic display [17]. The African Penguin has the nickname of “jackass” as it makes a donkey-like sound. In our study, this vocalisation was exclusively observed in the breeding season. We hypothesise that it served both to attract mates and as advertisement display of nest occupancy. Moreover, we observed that when a penguin performed the *ecstatic display song*, it was frequently followed by many other members of the colony in chorus. Conversely, the *mutual ecstatic song* was performed during the mutual ecstatic display [17], especially when a mate arrived at the nest. Partners often emitted this call simultaneously, overlapping in a duet. Specifically, mates stand facing each other with their wings held against or slightly away from their sides. We observed that many pairs also emitted this call as a threat towards penguins that came too close to their territory. Regarding begging vocalisations, we identified a *begging peep* emitted by chicks (<3 months of age) inside the nest, which probably has the function of stimulating food regurgitation by the parent. Finally, we detected a *begging moan* uttered by juveniles (3 to 12 months of age), which has not been previously reported in the literature, and is thus described here for the first time. At this age, penguins have not yet moulted for the first time and, therefore, they still have the characteristic juvenile plumage. During emission of this call, the juvenile bird stands up near a parent, places its beak perpendicular to the beak of its parent, and utters until it is fed. For this reason, we can state that this call still maintains a clear contextual use as a food request. However, it is important to note that acoustic features of this vocalisation have many more similarities with adult calls, in all the source-related parameters and energy quartiles (especially Q75%), than with *begging peeps* of chicks (Table 2). Moreover, the FM rate and AM rate values were similar to those measured on the adult *contact calls* (Table 2). These findings suggest complete development of the African Penguin vocal apparatus during the early months of life. Accordingly, Heath and Randall [43] observed that captive-reared chicks of this species can reach the body weight of the adults in approximately 120 days, with variations depending on the energy characteristics of the diet.

For each vocal signal, we measured 15 spectral and temporal acoustic descriptors that we used to perform a principal component analysis followed by classification of signals with a stepwise, cross-validated discriminant function analysis (DFA). The DFA correctly classified 90.50% of the penguins' calls according to the predicted vocal category previously identified by visual inspection of spectrograms. The accuracy we achieved is higher than that obtained in recent vocal classification studies in both birds (e.g. 83.3% obtained by Baldo and Mennill [44]), and mammals (e.g. 79.6% obtained by Barros *et al.* [45]; 69.1% obtained by Déaux and Clarke [46]). To date, this is the first study to provide acoustic measurements and statistical validation for the entire vocal repertoire of the African Penguin.

Jitter and Shimmer parameters were important factor loadings in PC2, and we measured the highest values in the *agonistic call* and *mutual display song* vocalisations. Both these vocalisations were uttered when a high level of arousal was present in the emitter. In particular, the first call type is produced in aggressive behavioural contexts, while the second is uttered both when members return to the nest and towards intruders in territorial clashes. Jitter is known to provide human listeners with cues about the utterer's affective state [38], and several authors have suggested that Jitter and Shimmer could be reliable indicators of the level of arousal in non-human mammals [39], [40]. Our findings demonstrate that these measurements could also be reliable indicators for detecting vocal types associated with behavioural contexts characterised by a high level of arousal in penguins.

The vocal categories we examined mostly correspond to those reported by Thumser and Ficken [18] in the repertoire of two captive colonies of African Penguin. However, these authors labelled calls with the terminology used by Boersma [47] to verbally describe vocalisations of wild Galapagos Penguins (*Spheniscus mendiculus*). In particular, for two vocal types for which acoustic measurements were performed by Thumser and Ficken [18], we found concordance for the *contact call* duration but not for the mean fundamental frequency. Concerning the *ecstatic display song*, we found compatible values for the total duration of the song, number of syllables, the duration of the longest syllable and mean fundamental frequency of the longest syllable. By contrast, we did not find a similar sum of the inter-syllable intervals as our average value was three times greater than that reported by Thumser and Ficken [18]. Finally, we identified a new type of syllable in the *ecstatic display song* (Figure 1c, indicated by arrow number 3) emitted during the inspiration phase. Playback experiments will be necessary to investigate whether this utterance has a biological significance or is just the result of an intense inhalation of air.

Although we cannot exclude that the list of calls in this studied colony may be incomplete (given that a captive environment has been proven to restrict the acoustic repertoire of animals [48]) it is highly likely that our classification is exhaustive for the vocal repertoire of free-living African Penguins. Eggleton and Siegfried [17] provided a verbal description of six different vocalisations in wild adult African Penguin. In our study, we found a correspondence for two of these six calls, namely the *ecstatic display song* and the *mutual display song*. However, we were unable to identify vocalisations that could be specifically assigned to the “aggressive barking”, “growling” and “aggressive braying” reported by this group, and keepers involved in the daily management of the colony confirmed this observation. These vocal categories were also not present in the studies of Thumser and Ficken [18] and Jouventin [13]. In the absence of spectrographic representations and quantitative acoustic measurements for comparison, we can only hypothesise, by the description of the behavioural contexts of emission, that these would merge into the *agonistic call*. The additional partitioning by Eggleton and Siegfried [17] could be the result of a subjective perception by different human listeners of the same call type heard in different agonistic contexts.

The source-related (F0) acoustic parameters measured in this study were the most important in discriminating between call types (PC1). However, we suggest, from observing the spectrograms (Figure 1), and from the heavy factor loadings (r>0.70) of the frequency quartiles that were grouped together in PC3 (Q25%, Q50% and Q75%), that a filter effect of the vocal tract may exist in the vocal output of this species. In particular, we observed that the values of the frequency quartiles vary according to the call type uttered. Accordingly, previous studies [12] have related the energy distribution to the mode of production of bird calls, showing that birds can use the pharyngeal constriction and inflection of the oesophagus to induce a modification of the energy distribution in the spectrum.

To date, the “two-voices” system [7], [14] in non-nesting species, and the pitch of the song and the relative values of harmonics in species that build nests [15] have been recognised as important acoustic cues for individual recognition in penguins [14]. Conversely, the “source-filter” theory of voice production [8], occasionally applied to birds [11], has never been extensively used to investigate whether acoustic cues of individuality, body size, gender or age could be encoded in penguin vocalisations. Further studies, to examine in detail the vocal behaviour of the African Penguin, from a source-filter perspective would be especially valuable. In particular, research efforts should be directed towards measuring formant frequencies [5], [11] in selected call types (particularly *contact call* and *display songs*), and evaluating whether individual variation in morphology and size of the vocal apparatus could result in individual acoustic distinctiveness [30]. Identifying reliable cues of vocal individuality in the African Penguin vocalisations would also be instrumental in developing technology for recognising and tracking wild penguins through emitted sounds, and estimating population sizes of this endangered species, whilst minimising any disturbance of the penguins. A recent study by Borker *et al.* [49] underlined the importance of vocal activity for studying large seabird colonies. In particular, they showed how the automated acoustic survey approach can both moderate biases common in standard survey approaches (e.g. collection of data by different observers), and even reduce costs in the monitoring of remote colonies.

In conclusion, this study (1) identifies and provides a statistical validation for six vocal categories in the repertoire of the African Penguin; (2) reports a new vocalisation (*begging moan*) used as a food request by juveniles towards parents, and a syllable emitted in the inspiration phase of the *ecstatic display song*, never previously described in the literature; (3) standardizes the terminology for the calls of this species; (4) suggests the use of the source-filter theory to further study the vocal communication in nest-building penguins of the genus *Spheniscus*.

## Supporting Information

Table S1 **Published studies on the vocal repertoire of the African Penguin.**
(PDF)

Video S1 ***Contact calls*** **uttered by adult African Penguins to maintain cohesion with colony members located out of visual range.**
(M4V)

Video S2 ***Agonistic call*** **uttered during fighting between two adults.**
(M4V)

Video S3 ***Ecstatic display song*** **uttered by a male in front of its nest, during the breading season.**
(M4V)

Video S4 ***Mutual display songs*** **made by a pair when one mate arrives at the nest.**
(M4V)

Video S5 ***Begging moans*** **of a juvenile (6 months old) uttered towards a parent. During emission, the juvenile performs a head shaking display.**
(M4V)

Video S6 ***Begging peeps*** **made by a chick (1 month old) at the nest. The calls and the head shaking stimulate food regurgitation by the parent.**
(M4V)

## References

[1] Alstroöm P, Ranft R (2003) The use of sounds in avian systematics, and the importance of bird sound archives. Bull Br Orn Club Supplement 123A: 114–135.

[2] Laiolo P (2010) The emerging significance of bioacoustics in animal species conservation. Biol Conserv 143: 1635–1645.

[3] Catchpole CK, Slater PJB (2008) Bird Song: Biological Themes and Variations. New York: Cambridge University Press. 335 p.

[4] Greenewalt CH (1968) Bird song: Acoustics and physiology. Washington, DC: Smithsonian Institution Press. 194 p.

[5] Fitch WT (1999) Acoustic exaggeration of size in birds via tracheal elongation: comparative and theoretical analyses. J Zool 248: 31–48.

[6] Goller F, Larsen ON (1997) A new mechanism of sound generation in songbirds. Proc Natl Acad Sci U S A 94: 14787–14791. 9405691 10.1073/pnas.94.26.14787 PMC25115

[7] Aubin T, Jouventin P, Hildebrand C (2000) Penguins use the two–voice system to recognize each other. P Roy Soc B-Biol Sci 267: 1081–1087. 10.1098/rspb.2000.1112 PMC1690651 10885512

[8] Fant G (1960) Acoustic Theory of speech production. The Hauge, Netherlands: Mouton & Co.

[9] Taylor AM, Reby D (2010) The contribution of source-filter theory to mammal vocal communication research. J Zool 280: 221–236.

[10] Cheng J, Xie B, Lin C, Ji L (2012) A comparative study in birds: call-type-independent species and individual recognition using four machine-learning methods and two acoustic features. Bioacoustics 21: 157–171.

[11] Budka M, Osiejuk TS (2013) Formant Frequencies are Acoustic Cues to Caller Discrimination and are a Weak Indicator of the Body Size of Corncrake Males. Ethology 119: 960–969.

[12] Riede T, Beckers GJL, Blevins W, Suthers RA (2004) Inflation of the esophagus and vocal tract filtering in ring doves. J Exp Biol 207: 4025–4036. 15498948 10.1242/jeb.01256

[13] Jouventin P (1982) Visual and Vocal Signals in Penguins, Their Evolution and Adaptive Characters. Adv Ethol 58 (S24) 3–148.

[14] Aubin T (2004) Penguins and their noisy world. An Acad Bras Cienc 76: 279–283. 15258640 10.1590/s0001-37652004000200015

[15] Jouventin P, Aubin T (2002) Acoustic systems are adapted to breeding ecologies: individual recognition in nesting penguins. Anim Behav 64: 747–757.

[16] Crawford RJM, Williams AJ, Hofmeyer JH, Klages NTW, Randall RM, et al (1995) Trends of African Penguin *Spheniscus demersus* populations in the 20^th^ century. Afr J Marine Sci 16: 101–118.

[17] Eggleton P, Siegfried WR (1977) Displays of the Jackass Penguin. Ostrich 50 (3) 139–167.

[18] Thumser NN, Ficken MS (1998) A comparison of the vocal repertoires of captive *Spheniscus* penguins. Marine Ornithology 26: 41–48.

[19] BirdLife International (2012) *Spheniscus demersus* In: IUCN 2012. IUCN Red List of Threatened Species. Version 2012.2. <www.iucnredlist.org>. Downloaded on 02 April 2013.

[20] Barham PJ, Crawford RJM, Underhill LG, Wolfaardt AC, Barham BJ, et al (2006) Return to Robben Island of African Penguins that were rehabilitated, relocated or reared in captivity following the Treasure oil spill of 2000. Ostrich 77: 202–209.

[21] Crawford RJM, Altwegg R, Barham BJ, Barham PJ, Durant JM, et al (2011) Collapse of South Africa's penguins in the early 21^st^ century. Afr J Mar Sci 33: 139–156.

[22] Blumstein DT, Mennill DJ, Clemins P, Girod L, Yao K, et al (2011) Acoustic monitoring in terrestrial environments using microphone arrays: applications, technological considerations and prospectus. J Appl Ecol 48: 758–767.

[23] Brandes TS (2008) Automated sound recording and analysis techniques for bird surveys and conservation. Bird Conserv Int 18: S163–S173.

[24] Stachowicz JB, Vannoni E, Pitcher BJ, Briefer EF, Geffen E, et al (2014) Acoustic divergence in the rut vocalizations of Persian and European fallow deer. J Zool 1: 1–9.

[25] Favaro L, Briefer EF, McElligott AG (2014) Artificial Neural Network approach for revealing individuality, group membership and age information in goat kid contact calls. Acta Acust united Ac 100 (4) 782–789 doi:10.3813/AAA.918758

[26] ASAB/ABS (2006) Guidelines for the treatment of animals in behavioural research and teaching. Anim Behav 71: 245–253. 10.1006/anbe.1999.1349 10640387

[27] WAZA (2005) Ethical Guidelines for the Conduct of Research on Animals by Zoos and Aquariums. 60^th^ Annual Conference of the World Association of Zoos and Aquariums, New York (USA). Available: http://www.waza.org/en/site/conservation/code-of-ethics-and-animal-welfare. Accessed 13 January 2014.

[28] Altmann J (1974) Observational study of behavior: sampling methods. Behaviour 49: 227–267. 4597405 10.1163/156853974x00534

[29] Boersma P, Weenink D (2013) Praat: doing phonetics by computer. http://www.praat.org.

[30] Reby D, McComb K (2003) Anatomical constraints generate honesty: acoustic cues to age and weight in the roars of red deer stags. Anim Behav 65: 519–530.

[31] Charlton BD, Zhihe Z, Snyder RJ (2009) Vocal cues to identity and relatedness in giant pandas (*Ailuropoda melanoleuca*). J Acoust Soc Am 126: 2721–2732. 19894848 10.1121/1.3224720

[32] Titze IR, Horii Y, Scherer R (1987) Some technical considerations in voice perturbation measurements. J Speech Lang Hear R 30: 252–260. 10.1044/jshr.3002.252 3599957

[33] Titze IR, Liang H (1993) Comparison of F0 extraction methods for high-precision voice perturbation measurements. J Speech Lang Hear R 36: 1120–1133. 10.1044/jshr.3606.1120 8114479

[34] Boersma P (2009) Should jitter be measured by peak picking or by waveform matching? Folia Phoniatr Logo 61 (5) 305–308. 10.1159/000245159 19828997

[35] Boersma P (2004) Stemmen meten met Praat. Stem-, Spraak- en Taalpathologie 12: 237–251.

[36] Brockmann M, Drinnan MJ, Storck C, Carding PN (2011) Reliable jitter and shimmer measurements in voice clinics: the relevance of vowel, gender, vocal intensity, and fundamental frequency effects in a typical clinical task. J Voice 25: 44–53. 20381308 10.1016/j.jvoice.2009.07.002

[37] Farrús M, Hernando J, Pascual P (2007) Jitter and Shimmer Measurements for Speaker Recognition. Proceedings of the international conference Interspeech 2007: 778–781.

[38] Bachorowski JA, Owren MJ (1995) Vocal expression of emotion: acoustic properties of speech are associated with emotional intensity and context. Psych Sci 6: 219–224.

[39] Li X, Tao J, Johnson MT, Soltis J, Savage A, et al (2007) Stress and emotion classification using jitter and shimmer features. Proceedings IEEE International Conference Acoustics, Speech, and Signal Processing 1081–1084.

[40] Briefer EF (2012) Vocal expression of emotions in mammals: mechanisms of production and evidence. J Zool 288: 1–20.

[41] Kaiser HF (1958) The varimax criterion for analytic rotation in factor analysis. Psychometrika 23: 187–200.

[42] Searcy WW, Nowicki S (2005) The Evolution of Animal Communication. Oxfordshire: Princeton University Press. 270 p.

[43] Heath RGM, Randall RM (1985) Growth of Jackass penguin chicks, *Spheniscus demersus*, hand reared on different diets. J Zool 205: 91–105.

[44] Baldo S, Mennill DJ (2011) Vocal behavior of Great Curassows, a vulnerable Neotropical bird. J Field Ornithol 82: 249–258.

[45] Barros KS, Tokumaru RS, Pedroza JP, Nogueira SSC (2011) Vocal Repertoire of Captive Capybara (*Hydrochoerus hydrochaeris*): Structure, Context and Function. Ethology 117: 83–93.

[46] Déaux ÉC, Clarke JA (2013) Dingo (*Canis lupus dingo*) acoustic repertoire: form and contexts. Behaviour 150: 75–101.

[47] Boersma PE (1976) An ecological and behavioral study of the Galapagos Penguin. Living Bird 15: 43–93.

[48] Midling K, Soldal AV, Fosseidengen JE, Øvredal JT (2002) Calls of the Atlantic cod: does captivity restrict their vocal repertoire? Bioacoustics 12: 233–235.

[49] Borker AL, McKown MW, Ackerman JT, Eagles-Smith CA, Tershy BR, et al (In Press) Vocal activity as a low cost and scalable index of seabird colony size. Conserv Biol doi:10.1111/cobi.12264 10.1111/cobi.12264 24628442

