# Supplementary material for: The Vocal Repertoire of the African Penguin (Spheniscus demersus): Structure and Function of Calls
Source: PLoS One. 2014 Jul 30;9(7):e103460. doi: 10.1371/journal.pone.0103460 (PMC4116197; doi:10.1371/journal.pone.0103460)
Supplement: Table S1 — Published studies on the vocal repertoire of the African Penguin. (PDF) [file pone.0103460.s001.pdf]

Table S1. Published studies on the vocal repertoire of the African Penguin (*Spheniscus demersus*).

Author(s)	Setting (wild/captive)	Number of penguins	Period of observation	Vocalisation	Behavioural context(s)	Acoustic measurements	Spectrographic representation
Eggleton & Siegfried (1977)	Wild	130	Year round	*Ecstatic display song*	Territorial defence. This call was also suggested to signal availability for pairing and to communicate individual identity	No	No
				Aggressive braying	Aggression	No	No
				Hissing	Threat: emitted by chicks and juveniles towards external adults that approach the nest	No	No
				Aggressive barking	Aggression: occurred between moulting juveniles and adults	No	No
				Growling	Aggression: emitted simultaneously with pecking behaviour	No	No
				Mutual display song	Reunion between members of the pair. This call was also observed as a stimulation to begging from parents to chicks	No	No
Jouventin (1982)	N/A	N/A	N/A	*Ecstatic display song*	N/A	No	Yes
	Wild	5	N/A	*Contact call*	Visual isolation: for maintaining acoustic contact when floating on the sea surface	No	No
	N/A	N/A	N/A	*Mutual display song*	N/A	No	Yes
Thumser *et al*. (1996)	Captive	12	Breeding season	*Bray call*	Territorial defence but also suggested as signalling availability for pairing	Yes	No

Thumser & Ficken (1998)	Captive 15	Breeding	season	*Bray call*	Territorial defence but also for attracting partners	Yes	Yes
				Haw	Visual isolation: emitted to identify penguins' position	Yes	Yes
				Yell	Aggression: emitted during agonistic encounters	No	No
				Throb	Emitted by pairs in the nest	No	No
				Peep	Uttered by chicks in the nest as a food request	No	No

Note: N/A = information not available.

References

Eggleton P, Siegfried WR. (1977) Displays of the Jackass Penguin. Ostrich 50 (3): 139-167.

Jouventin P. (1982) Visual and Vocal Signals in Penguins, Their Evolution and Adaptive Characters. Adv Ethol 58 (S24): 3-148.

Thumser NN, Karron JD, Ficken MS. (1996) Interspecific variation in the call of *Spheniscus* penguins. Wilson Bulletin 108: 72-79.

Thumser NN, Ficken MS. (1998). A comparison of the vocal repertoires of captive *Spheniscus* penguins. Marine Ornithology 26: 41-48.
